# FOXP3 gene polymorphism is associated with hepatitis B-related hepatocellular carcinoma in China

**DOI:** 10.1186/1756-9966-32-39

**Published:** 2013-06-10

**Authors:** YanHui Chen, HengHui Zhang, WeiJia Liao, JinXue Zhou, GaiXia He, XingWang Xie, Ran Fei, LiLing Qin, Lai Wei, HongSong Chen

**Affiliations:** 1 Peking University People’s Hospital, Peking University Hepatology Institute, No.11 Xizhimen South Street, Beijing 100044, China; 2 Beijing Key Laboratory of Hepatitis C and Immunotherapy for Liver Diseases, Beijing 100044, P. R. China; 3 Affiliated Hospital of Guilin Medical University, Guilin 541004, P. R. China; 4 Department of Hepatobiliary and Pancreatic Surgery, Henan Tumor Hospital, Zhengzhou 450008, P. R. China

**Keywords:** Carcinoma, Hepatocellular, Hepatitis B, Chronic, Gene polymorphism, FOXP3

## Abstract

**Background:**

Previous evidence has shown that the FOXP3 gene was involved in the pathogenesis of several tumors; however, the correlation between single nucleotide polymorphisms (SNPs) in the FOXP3 gene and the susceptibility to hepatitis B-related hepatocellular carcinoma (HCC) remains unclear.

**Methods:**

We analyzed two SNPs in the FOXP3 gene, rs2280883 and rs3761549, in 392 patients with HCC, 344 patients with chronic hepatitis B (CHB) and 372 matched healthy controls. Genotyping was performed by MALDI-TOF Mass Spectrometry for all donors.

**Results:**

Compared to healthy controls, HCC patients had higher frequencies of the TT genotype (79.6%) at rs2280883 and the CC genotype (77.6%) at rs3761549 of the FOXP3 gene; CHB patients also had higher frequencies of the TT genotype (74.1%) at rs2280883 and the CC genotype (74.6%) at rs3761549. There were no significant differences in the distribution of FOXP3 genotypes between CHB donors and HCC donors. The TT genotype at rs2280883 was more frequent in patients with HCC than healthy donors (*P* = 0.01), but no significant difference was observed in this genotype between CHB and healthy donors (*P* = 0.479). C allele frequency at rs3761549 was higher in HCC patients than healthy donors (*P* = 0.03), but distribution of this allele was not significantly different between CHB patients and healthy donors (*P* = 0.11). Stratified analysis showed that the CC genotype at rs3761549 was significantly associated with a high incidence of portal vein tumor thrombus (*P* = 0.02) and that the TT/CT genotype at rs3761549 was significantly associated with an increased rate of tumor recurrence in HCC patients (*P* = 0.001).

**Conclusions:**

Our results suggested that the FOXP3 gene polymorphisms at rs2280883 and rs3761549 may be associated with hepatitis B-related HCC. At rs3761549, the CC genotype and the TT/CT genotype were associated with a high incidence of portal vein tumor thrombus and tumor recurrence, respectively.

## Background

Hepatocellular carcinoma is the sixth most common malignancy and the third most common cause of cancer-related death worldwide [1], but the disease progression of HCC remains poorly understood. A previous study showed that the local tumor immune microenvironment plays an important role in cancer suppression and promotion and that one of the main factors leading to tumor immune tolerance in the local tumor microenvironment is the influence of CD4+/CD25+/FOXP3+ regulatory T cells (Tregs) [2]. The number of Tregs increases in response to infection by pathogenic microorganisms, including the hepatitis B virus; this increase inhibits CD4+ and CD8+ T-cell activation, proliferation and cytokine secretion, thus affecting the host immune response to infection and leading to chronic infection [3-6]. This phenomenon indicates that the FOXP3 gene may play a role in inflammation and chronic infections such as hepatitis B, which may increase the risk of carcinoma.

FOXP3 is a specific molecular marker of Tregs that plays an important role in the development of Tregs and their inhibitory functions [7,8]. Increased levels of FOXP3+ Tregs in the peripheral blood and tumor tissue have been reported in patients with various types of cancer, including ovarian [9,10], breast [11], hepatocellular carcinoma [12] and other tumors [13]; the accumulation of Tregs in local lymph nodes or in tumors is associated with a less favorable prognosis [9-14]. Although Tregs are the major cell type expressing FOXP3, it has recently been demonstrated that the tumor cell itself can express FOXP3, such as pancreatic cancer [15], melanoma [16] and other tumor types [17], and the function of FOXP3 may represent a new mechanism of immune evasion in cancers.

It has been confirmed that genetic polymorphisms in FOXP3 are related to the development of autoimmune diseases, such as allergic rhinitis [18], idiopathic infertility and endometriosis-related infertility [19]. These results suggested that polymorphisms at rs2280883 within the FOXP3 gene may be associated with idiopathic infertility, while polymorphisms at rs3761549 may be related to endometriosis. However, it remains unclear whether FOXP3 gene polymorphism is associated with hepatitis B-related HCC.

Based on FOXP3 gene SNP genotype data from the HapMap Phase II + Phase III database, two tagSNPs, rs2280883 and rs3761549, were selected for genotyping because these two SNPs could cover 80% of the MAF > 0.1 SNPs. To investigate the correlation between specific SNPs in the FOXP3 gene and hepatitis B-related HCC, Matrix-Assisted Laser Desorption/Ionization Time of Flight (MALDI-TOF) Mass Spectrometry was used to screen for the presence of the FOXP3 gene polymorphisms in HCC donors, CHB donors and healthy donors. Here, we present data describing an association between FOXP3 genetic variation and susceptibility to hepatitis B-related HCC in all donors.

## Materials and methods

### Study subjects and peripheral blood samples

Peripheral blood samples were obtained from 392 HCC patients, 344 CHB patients and 372 healthy donors. HCC patients were treated at the Guilin Medical University-affiliated hospital between November 2001 and April 2010. CHB patients with diagnoses conformed to the latest diagnostic criteria [20] were from the Peking University Hepatology Institute (Peking, China) between November 2001 and April 2010. Healthy donors were patients undergoing routine physical examination at Peking University People’s Hospital. General patient information was recorded in detail, including age, gender, alcohol abuse, cirrhosis, presence of hepatitis B or hepatitis C virus (HCV) infection, alpha-fetoprotein (AFP), alanine aminotransferase (ALT), aspartate aminotransferase (AST), γ-glutamyl transpeptidase (GGT) and total bilirubin (TBIL) levels; this information is provided in Table 1. HCC patient information, such as primary tumor size, histologic tumor type, histologic grade, lymph node (LN) stage, portal vein thrombosis and distant metastasis, were routinely assessed according to the TNM staging criteria proposed in 2002 by the International Union against Cancer (UICC) and American Joint Committee on Cancer (AJCC). All CHB patients have been screened by B-ultrasound and CT examination to exclude cancers. Healthy donors were selected at random; none had HBV and HCV infection according to screening for HBsAg and anti-HCV, and donors with liver cirrhosis or tumor-related diseases were excluded by B-ultrasound and CT examination. The study was implemented after receiving the approval of the Medical Ethics Committee of Peking University People’s Hospital. Written informed consent was obtained from all patients prior to sample collection according to the Declaration of Helsinki in 1995 (as revised in Tokyo, 2004).

**Table 1:** Demographic, clinical and biological characteristics of all donors

**Characteristics**		**HCC n(%)**	**CHB n(%)**	**HEAL n(%)**	***P *value**
		**n = 392**	**n = 344**	**n = 372**	
Age	<55	252(64.3)	223(64.8)	245(65.9)	0.90
	≥55	140(35.7)	121(35.2)	127(34.1)	
Gender	Male	345(88.0)	267(77.6)	306(82.3)	**0.001**
	Female	47(12.0)	77(22.4)	66(17.7)	
Alcohol abuse	Absent	75(19.1)	44(12.8)	31(8.3)	**<0.001**
	Present	317(80.9)	300(87.2)	341(91.7)	
Cirrhosis	Absent	50(12.8)	331(96.2)	372	
	Present	342(87.2)	13(3.8)	0	
Anti-HCV positive		0	0	0	
HBsAg positive		364(92.9)	344(100.0)	0	
AFP(ng/ml)		923.3 ± 597.1	7.6 ± 6.9		**<0.001**
ALT(IU/L)		51.0 ± 24.0	54.0 ± 41.0	21.0 ± 8.1	0.30
AST(IU/L)		36.3 ± 29.4	45.3 ± 34.3	26.1 ± 6.9	0.67
GGT(IU/L)		27.7 ± 23.5	39.4 ± 35.7	19.5 ± 17.1	0.56
TBIL(μmol/L)		16.4 ± 12.6	19.0 ± 7.3	12.1 ± 4.2	0.56

Alanine aminotransferase (ALT), aspartate aminotransferase (AST), γ-glutamyl transpeptidase (GGT) and total bilirubin (TBIL), Alpha Fetoprotein (AFP).

### FOXP3 SNP genotyping

FOXP3 gene SNP genotype data were retrieved from the HapMap Phase II + Phase III database, and the haplotypes were analyzed with restrictive standards (r2 > 0.8 and a minimum allele frequency (MAF) > 0.1) in Haploview 4.2 software. Finally, two tagSNPs, rs2280883 and rs3761549, which were able to cover 80% of the MAF > 0.1 SNPs, were selected for genotyping. DNA was extracted from the peripheral blood samples from each patient using standard methods. Genotyping was performed by MALDI-TOF Mass Spectrometry for all donors. The DNA from the donors was blinded, coded and tested using a 384-well format SpectroCHIP microarray. PCR primers and single base extension primers for rs2280883 and rs3761549 were designed using Sequenom Assay design 3.1 software. These primer sequences are shown in Table 2. A MALDI-TOF Mass Spectrometer was used for data acquisition from the SpectroCHIP. The results were analyzed using Sequenom MassARRAY RT software.

**Table 2:** Sequences of PCR primers and single-base extension primers

**SNPs**	**PCR primers sequences**	**Single base extension primers sequences**
rs2280883	F: ACGTTGGATGAGATGAAGGAGTTGGGATGG	GACAAGGAAAGGTTGGGAA
	R: ACGTTGGATGTGTCAATACACCCCCAACTG	
rs3761549	F: ACGTTGGATGACCCCACAGGTTTCGTTCC	AGTTTCGTTCCGAGAACT
	R: ACGTTGGATGACATCACCTACCACATCCAC	

“F”: Forward, “R”: Reverse.

### Statistical analysis

ALT, AST, GGT, TBIL and AFP levels were reported as the mean ± standard deviation, and the distributions of these variables were compared by Kruskal-Wallis tests; AFP values between HCC and CHB donors were compared by the Mann–Whitney U test. Patients’ age, gender, alcohol abuse and genotype frequencies were obtained by direct counting and statistical analysis was performed by the chi-squared test. Odds of allele and genotype with 95% confidence intervals (95% CI) in patients with HCC versus CHB or healthy donors were also calculated. P-values less than 0.05 were considered statistically significant. SPSS 13.0 for Windows was used for all statistical calculations.

## Results

### The distribution of demographic and clinical characteristics of all donors

As shown in Table 1, there were no significant differences in the age, AST, ALT, GGT, and TBIL levels between either HCC or CHB groups and the healthy group (all *P* > 0.05). HBsAg was undetected in all healthy donors, but 92.9% of HCC patients were HBsAg positive; all these donors were anti-HCV negative. The AFP level and the frequency of cirrhosis were significantly higher in the HCC group than in the CHB group. The distributions of gender and alcohol abuse showed that male alcohol-abusing donors accounted for the majority of the HCC, CHB and healthy donors. All donors were placed into the three groups based on the principle of random sampling; these characteristics showed the true representative natural history of the incidence of CHB and HCC in China.

### The analysis of FOXP3 SNPs allele frequency in all donors

In Table 3, there was no significant difference in the distribution of C and T alleles at rs2280883 of FOXP3 between HCC and healthy donors (*P* = 0.20), and the frequencies of C and T alleles at rs2280883 were similar in CHB donors and healthy donors (*P* = 0.54). The C allele frequency at rs3761549 was higher in HCC donors than in healthy donors (OR 1.32; 95% CI 1.03-1.70; *P* = 0.03), but there was no significant difference in the distribution of C and T alleles at rs3761549 between CHB patients and healthy donors (*P* = 0.11). The differences in allele frequencies at rs2280883 and rs3761549 were not observed between CHB donors and HCC donors (*P* = 0.06, *P* = 0.58). These results showed that the C allele at rs3761549 was associated with HCC but not with CHB.

**Table 3:** The analysis of FOXP3 SNPs allele frequency and genotype in all donors

**SNPs**	**HCC n(%)**	**CHB n(%)**	**HEAL n(%)**	**HCC-HEAL**		**CHB-HEAL**		**HCC-CHB**	
	**n = 392**	**n = 344**	**n = 372**	**OR(95% CI)**	***P *value**	**OR(95% CI)**	***P *value**	**OR(95% CI)**	***P *value**
Allele									
rs2280883					0.20		0.54		0.06
C	134(17.1)	144(20.9)	146(19.6)	0.84(0.65-1.10)		1.07(0.87-1.31)		0.78(0.60-1.01)	
T	650(82.9)	544(79.1)	598(80.4)	1.18(0.91-1.54)		0.98(0.93-1.04)		1.28(0.99-1.67)	
rs3761549					**0.03**		0.11		0.58
C	630(81.2)	549(80.0)	554(76.5)	1.32(1.03-1.70)		1.05(0.99-1.11)		1.08(0.83-1.40)	
T	146(18.8)	137(20.0)	170(23.5)	0.76(0.59-0.97)		0.85(0.70-1.04)		0.93(0.72-1.20)	
Genotype									
rs2280883					**<0.001**		**<0.01**		0.158
CC	54(13.8)	55(16.0)	41(11.0)	1.29(0.84-1.99)		1.54(0.99-2.37)		0.84(0.56-1.26)	
TT	312(79.6)	255(74.1)	267(71.8)	1.53(1.10-2.14)		1.13(0.81-1.57)		1.38(0.98-1.95)	
CT	26(6.6)	34(9.9)	64(17.2)	0.34(0.21-0.55)		0.53(0.34-0.82)		0.65(0.38-1.10)	
rs3761549					**<0.001**		**<0.001**		0.239
CC	301(77.6)	256(74.6)	233(64.4)	1.92(1.39-2.64)		1.63(1.18-2.25)		1.18(0.84-1.65)	
TT	59(15.2)	50(14.6)	41(11.3)	1.40(0.92-2.15)		1.34(0.86-2.08)		1.05(0.70-1.58)	
CT	28(7.2)	37(10.8)	88(24.3)	0.24(0.15-0.38)		0.38(0.25-0.57)		0.64(0.39-1.08)	

“HEAL”: Healthy donors.

### The association between FOXP3 genotype and susceptibility to hepatitis B-related HCC

For HCC and healthy donors, the TT genotype at rs2280883 (OR 1.55; 95% CI 1.11-2.17; *P* = 0.01) was more frequent in patients with HCC than the CC or CT variants, but the frequency of the CC genotype was not significantly different between HCC and healthy donors (*P* = 0.249); at rs3761549, the CC genotype (OR 1.92; 95% CI 1.39-2.64; *P* < 0.001) was more frequent in patients with HCC than the TT or CT variants, but the frequency of the TT genotype was not significantly different between HCC and healthy donors (*P* = 0.118). Compared to HCC patients, the CT genotype at both rs2280883 and rs3761549 was significantly more frequent in healthy donors than the CC or TT variants (both *P* < 0.001) (Table 3 and Additional file 1: Table S1). For CHB donors and healthy donors, the CT genotype at rs2280883 was more frequent in healthy donors than the CC or TT variants (*P* = 0.004), but there were no significant differences in the distribution of either CC or TT genotypes between CHB donors and healthy donors (*P* = 0.051, *P* = 0.479); at rs3761549, the CC genotype (OR 1.63; 95% CI 1.18-2.25; *P* = 0.003) was more frequent in patients with CHB than the TT or CT variants, but there was no significant difference in the distribution of the TT genotype between CHB donors and healthy donors (*P* = 0.198). The CT genotype was more frequent in healthy donors than the CC and TT variants (*P* < 0.001) (Table 3 and Additional file 1: Table S1). However, there were no significant differences in the FOXP3 genotype distribution between HCC donors and CHB donors at either rs2280883 or rs3761549 (Table 3 and Additional file 1: Table S1). Compared to healthy donors, the TT genotype at rs2280883 was more frequent in patients with HCC, but this genotype frequency was not significantly different between CHB and healthy donors, in addition, the CC genotype at rs2280883 was more frequent in CHB patients (16.0%) than in HCC patients (13.8%), but the TT genotype was more frequent in HCC patients (79.6%) than in CHB patients (74.1%); these results showed that the TT genotype at rs2280883 was associated with HCC but not with CHB.

### The stratified analysis of the association between FOXP3 genotypes and HCC clinical pathology variables

Because the FOXP3 genetic variants rs2280883 and rs3761549 were significantly associated with susceptibility to HCC, further analysis was performed to determine the relationship between the FOXP3 genotype and multiple HCC clinical pathology variables, such as age, gender, alcohol abuse history, tumor size, tumor nodule, tumor grade, lymph node metastasis, portal vein tumor thrombus, distant metastasis and recurrence. Follow-up records had not been completed for all of the patients, and detailed clinical pathology variables were available for only 188 cases; these details are shown in Table 4. The CC genotype of rs3761549 was more frequent in HCC patients with portal vein tumor thrombus (*P* = 0.02), and the TT and CT genotypes were more common in patients with recurrent HCC (*P* = 0.001).

**Table 4:** The analysis of the association between FOXP3 genotypes and HCC clinical pathology variables

**Characteristics**	**rs2280883**		***P *values**	**rs3761549**		***P *values**
	**TT n(%)**	**CC/CT n(%)**		**CC n(%)**	**TT/CT n(%)**	
	**n = 151**	**n = 37**		**n = 155**	**n = 33**	
Age			0.48			0.15
<55	101(66.9)	27(73.0)		109(70.3)	19(57.6)	
≥55	50(33.1)	10(27.0)		46(29.7)	14(42.4)	
Gender			0.216			0.33
Male	136(90.0)	30(81.1)		139(89.7)	27(81.8)	
Female	15(10.0)	7(18.9)		16(10.3)	6(18.2)	
Alcohol abuse			0.63			0.80
Absent	72(47.7)	16(43.2)		76(49.0)	17(51.5)	
Present	79(52.3)	21(56.8)		79(51.0)	16(48.5)	
Tumor Size (cm)			0.61			0.64
≤5	42(27.8)	9(24.3)		44(28.4)	7(21.2)	
>5, ≤10	57(37.7)	11(29.7)		54(34.8)	14(42.4)	
>10, ≤20	43(28.5)	14(37.9)		48(31.0)	9(27.3)	
>20	9(6.0)	3(8.1)		9(5.8)	3(9.1)	
Tumor nodule (No.)			0.54			0.48
1	98(64.9)	26(70.3)		104(67.1)	20(60.6)	
≥2	53(35.1)	11(29.7)		51(32.9)	13(39.4)	
Tumor grade			0.69			0.87
I	24(15.9)	3(8.1)		24(15.5)	3(9.1)	
II	24(15.9)	6(16.2)		24(15.5)	6(18.2)	
III	97(64.2)	27(73.0)		101(65.2)	23(69.7)	
IV	6(4.0)	1(2.7)		6(3.8)	1(3.0)	
lymph node metastasis			0.76			0.93
Absent	138(91.4)	35(94.6)		142(91.6)	31(93.9)	
Present	13(8.6)	2(5.4)		13(8.4)	2(6.1)	
portal vein tumor thrombus			0.76			**0.02**
Absent	119(78.8)	30(81.1)		118(76.13)	31(93.94)	
Present	32(21.2)	7(18.9)		37(23.87)	2(6.06)	
Distant Metastasis			0.59			0.73
Absent	136(90.1)	35(94.6)		142(91.6)	29(87.9)	
Present	15(9.9)	2(5.4)		13(8.4)	4(12.1)	
Recurrence			0.60			**0.001**
Absent	112(74.2)	29(77.4)		124(80.0)	17(51.5)	
Present	39(25.8)	8(21.6)		31(20.0)	16(48.5)	

## Discussion

FOXP3 is an accurate marker of primary Tregs in patients with immune-related disease and cancer [21]. Recently, it was shown that FOXP3 is not only expressed in Tregs but also in tumor cells of cancer patients; its expression level and function may represent a new mechanism of immune evasion in cancers [15-17]. Polymorphisms of the FOXP3 gene may change FOXP3 quantitatively or functionally, thereby contributing to an immune imbalance in cancer. To date, polymorphisms in the FOXP3 gene have been associated with a variety of immune-related diseases, such as allergic rhinitis [18], idiopathic infertility and endometriosis-related infertility [19]. However, there are no relevant reports on the relationship between FOXP3 gene polymorphism and cancer. Our study aimed to evaluate the association between FOXP3 gene polymorphisms and hepatitis B-related HCC. The results showed that the rs2280883 polymorphism was associated with HCC. Rs2280883, located in intron 9 very near a conserved gene transcription region of FOXP3, could cause splicing downstream, resulting in a less functional gene. The rs3761549 polymorphism was also significantly associated with HCC. The rs3761549 microsatellite, located in the promoter region of the gene, could theoretically affect gene expression, resulting in FOXP3 mRNA instability. These potential mechanisms need to be explored.

Based on our study, 92.9% of HCC patients have HBV infection; it is important to evaluate the association between FOXP3 gene polymorphism and CHB. Our data showed that there were also significant differences in FOXP3 genotype frequencies between CHB donors and healthy controls; both rs2280883 and rs3761549 polymorphisms were related to CHB, but there were no significant differences in FOXP3 genotype frequencies between CHB donors and HCC donors at either SNP. These results may suggest that the FOXP3 gene is involved in both inflammation and tumor pathogenesis or just the process of inflammation leading to neoplastic transformation; in contrast, nearly all HCC patients also had hepatitis B, so FOXP3 polymorphism may create a predisposition to CHB and cirrhosis, with HCC just a result of this predisposition. We found that the TT genotype at rs2280883 was more frequent in HCC patients than in CHB patients compared to healthy donors; this result suggested that the TT genotype at rs2280883 may be associated with HCC but not with CHB.

It has previously been reported that high levels of FOXP3 protein expression are associated with a poor prognosis and low survival of breast cancer [22]. Whether in Tregs or in tumor cells, FOXP3 expression plays an immunosuppressive role at the tumor site [15-17,23]. Taking into account these results for FOXP3 gene function, further analysis showed that the CC genotype at rs3761549 of FOXP3 was significantly more frequent in HCC patients with portal vein tumor thrombus, while the TT and CT genotypes were significantly more common in those patients with recurrence. These results may indicate that FOXP3 has a similar immunosuppressive effect in liver cancer as in other previously reported cancers. In addition, it would be interesting to see portal vein thrombosis incidence in hepatitis B-related HCC patients in the future; it is possible that this relationship between FOXP3 rs3761549 genotype and portal vein thrombosis may hold true and is related to Hepatitis B virus infection and not HCC itself.

The correlation between FOXP3 gene polymorphisms and HCV infection is also worth exploring. A previous study indicated that the microsatellite polymorphisms of the promoter/enhancer region of FOXP3 were not associated with chronic HCV infection [24], and in our study, we did not receive hepatitis C patients or hepatitis C-related HCC patients, preventing our discussion of FOXP3 gene polymorphisms in HCV infection. Current studies have rarely reported concrete relevance for FOXP3 expression in tumors; the transcript types and biological significance of FOXP3 in cancer remains unclear. Because of the complex relationship between inflammation and a tumor and the important role of FOXP3 in this relationship, it is difficult to clearly describe the relevance between FOXP3 gene polymorphisms and CHB or hepatitis B-related HCC.

Overall, our study showed that FOXP3 gene polymorphisms are related to hepatitis B-related HCC. Polymorphisms at rs2280883 and rs3761549 in the FOXP3 gene may be associated with CHB and HCC. The existence of HCC may be related to long-term inflammation due to CHB. Therefore, more in-depth studies should be conducted with more samples from a broader population to further elucidate the molecular mechanism by which FOXP3 affects the development of HCC.

## Abbreviations

SNPs: Single nucleotide polymorphisms; HCC: Hepatocellular carcinoma; CHB: Chronic hepatitis B; HBV: Hepatitis B virus; HCV: Hepatitis C virus; AFP: Alpha-fetoprotein; ALT: Alanine aminotransferase; AST: Aspartate aminotransferase; GGT: γ-Glutamyl transpeptidase; TBIL: Total bilirubin; MALDI-TOF: Matrix-assisted laser desorption/ionization time of flight; OR: Odds ratio.

## Competing interests

The authors declared that they have no competing interest.

## Authors’ contributions

YHC, HHZ and HSC contributed to the conception and design of the study. YHC and HHZ performed the statistical analysis and drafted and revised the manuscript. JXZ and WJL collected blood samples. GXH and RF performed the technical experiments. XWX interpreted the molecular analyses. LLQ collected blood samples and clinical information. LW participated in the design of the study and collected the clinical information. All authors read and approved the final version of the manuscript.

## Supplementary Material

Additional file 1: Table S1 The analysis of FOXP3 SNPs genotypes in all donors. The 2 × 2 tables were used for two comparisons of genotypes respectively in HCC patients or CHB patients versus healthy donors, to get accurate individual P-values. Click here for file
