# Supplementary material for: FOXP3 gene polymorphism is associated with hepatitis B-related hepatocellular carcinoma in China
Source: J Exp Clin Cancer Res. 2013 Jun 10;32(1):39. doi: 10.1186/1756-9966-32-39 (PMC3718693; doi:10.1186/1756-9966-32-39)
Supplement: Additional file 1: Table S1 — The analysis of FOXP3 SNPs genotypes in all donors. The 2 × 2 tables were used for two comparisons of genotypes respectively in HCC patients or CHB patients versus healthy donors, to get accurate individual P-values. [file 1756-9966-32-39-S1.pdf]

Supporting data:

Table s1. The analysis of FOXP3 SNPs genotypes in all donors

Genotype	HCC n(%)	CHB n(%)	HEAL n(%)	HCC-HEAL		CHB-HEAL		HCC-CHB	
	n=392	n=344	n=372	OR(95%CI)	P value	OR(95%CI)	P value	OR(95%CI)	P value
rs2280883									
CC	54(13.8)	55(16.0)	41(11.0)	1.29(0.84-1.99)	0.249	1.54(0.99-2.37)	0.051	0.84(0.56-1.26)	0.339
TT+CT	338(86.2)	289(84.0)	331(89.0)	0.78(0.50-1.20)		0.65(0.42-1.01)		1.19(0.79-1.79)	
TT	312(79.6)	255(74.1)	267(71.8)	1.55(1.11-2.17)	**0.01**	1.13(0.81-1.57)	0.479	1.38(0.98-1.95)	0.068
CC+CT	79(20.2)	89(25.9)	105(28.2)	0.64(0.46-0.90)		0.89(0.64-1.24)		0.73(0.51-1.03)	
CT	26(6.6)	34(9.9)	64(17.2)	0.34(0.21-0.55)	**<0.001**	0.53(0.34-0.82)	**0.004**	0.65(0.38-1.10)	0.108
CC+TT	366(93.4)	310(90.1)	308(82.8)	2.93(1.81-4.73)		1.90(1.21-2.96)		1.54(0.91-2.63)	
rs3761549									
CC	301(77.6)	256(74.6)	233(64.4)	1.92(1.39-2.64)	**<0.001**	1.63(1.18-2.25)	**0.003**	1.18(0.84-1.65)	0.351
TT+CT	87(22.4)	87(25.4)	129(35.6)	0.52(0.38-0.72)		0.61(0.44-0.85)		0.85(0.61-1.20)	
TT	59(15.2)	50(14.6)	41(11.3)	1.40(0.92-2.15)	0.118	1.34(0.86-2.08)	0.198	1.05(0.70-1.58)	0.812
CC+CT	329(84.8)	293(85.4)	321(88.7)	0.71(0.47-1.09)		0.75(0.48-1.17)		0.95(0.63-1.43)	
CT	28(7.2)	37(10.8)	88(24.3)	0.24(0.15-0.38)	**<0.001**	0.38(0.25-0.57)	**<0.001**	0.64(0.39-1.08)	0.091
CC+TT	360(92.8)	306(89.2)	274(75.7)	4.13(2.62-6.50)		2.66(1.75-4.03)		1.56(0.93-2.60)	

“HEAL”: Healthy donors.
